# Shape and joint angle data for seven European horse breeds and their repeatability

**DOI:** 10.1016/j.dib.2024.110799

**Published:** 2024-08-03

**Authors:** Annik Imogen Gmel, Luis P. Lamas, Teresa V. Rosa, Monika Stefaniuk-Szmukier, Weronika Klecel, Tamara Martin-Gimenez, Antonio Cruz, Michael A. Weishaupt, Markus Neuditschko

**Affiliations:** a Agroscope, Animal GenoPhenomics, Route de la Tioleyre 4, 1725 Posieux, Switzerland; b Equine Department, Vetsuisse Faculty, University of Zurich, Winterthurerstrasse 260, 8053 Zurich, Switzerland; c CIISA, Faculdade de Medicina Veterináriada Universidade de Lisboa, Lisboa, Portugal; d National Research Institute of Animal Production, University of Agriculture in Krakow, Krakow, Poland; e Department of Animal Genetics and Conservation, Institute of Animal Sciences, Warsaw University of Life Sciences, Warsaw, Poland; f Klinik für Pferdechirurgie und Orthopädie, Justus-Liebig Universität Giessen, Frankfurterstrasse 108, 39352 Giessen, Germany; g Universidad de Zaragoza, Zaragoza, Spain

**Keywords:** Equid, Geometric morphometrics, Imaging, Conformation

## Abstract

Conformation traits are important in the selection and distinction between horse breeds, but tend to be evaluated subjectively within a breed and cannot be compared between them. The horse shape space model, using a combination of 253 landmarks and semi-landmarks, provides objective information on the shape of a horse photographed from the side that can be compared between breeds. In this dataset, we are providing the full set of 253 landmarks for 1241 horses from seven breeds, including an R code file to extract joint angle information and transform the raw data into csv files for further analysis, such as breed comparisons, heritability or genome-wide association studies (single- or multibreed). The repeatability of the joint angles are also reported.

Specifications Table

**Table:** 

Subject	Veterinary Science
Specific subject area	Full-body geometric morphometric data of live, standing horses
Data format	Raw, Analyzed
Type of data	Tables, tps coordinate files
Data collection	Landmarks were collected from photographs following the landmark placement of Gmel et al. [1]. Each photograph contains a horse from the side view, in open posture, and was digitised by the same person using the open source program tpsDig2 [2], after creating a raw tps file from the photograph using tpsUtil [3]. For each breed, a subset of photographs has been digisited three times to assess repeatability of the digitising process.
Data source location	Institution: Agroscope Country: Switzerland
Data accessibility	Repository name: Mendeley Data identification number: DOI: 10.17632/ybvcdmtrpc.1 Direct URL to data: https://data.mendeley.com/datasets/ybvcdmtrpc/1

## 1 Value of the Data

• These data highlight morphological differences (conformation) in different horse breeds objectively.
• These data can be used to compare the conformation with data from additional horses from the same or additional breeds when using the same methodology.
• These data can also be used as phenotype in genetic studies (heritabilities, genome-wide association studies).

## 2 Background

The domestic horse shows a wide range of morphological variation within and across breeds. Unfortunately, with the exception of length measurements, morphological studies have mainly concentrated on judge scores from breeding competitions, which are subjective and cannot be compared between breeds [4]. Since 2015, a new model using geometric morphometrics, the horse shape space, has been available and consists of a set of 246 landmarks (lm) and semi-landmarks (slm) [5]. With the shape space model, it was possible to visualise judge bias in Lipizzaner horses for the first time [6]. Gmel et al. [1] increased the landmarks to 253 to study differences in joint angles and showed that breeding associations might benefit from using joint angles provided by the horse shape space that had higher heritabilities than the same trait assessed using a linear profiling scale. Several studies on the genetic architecture of conformation traits derived from the horse shape space model have been published on Lipizzaner (LIP) [7] and Franches-Montagnes (FM) horses [8]. Considering the increasing interest in geometric morphometrics in other horse breeds (e.g. [9]), this data article describes all data collected in LIP, FM and five additional European horse breeds, i.e. European Warmblood (WB), Shagya Arabians (SHA), Purebred Arabian (AR), Lusitanos (LUS), and Pura Raza Español (PRE).

## 3 Data Description

**Fig. 1:**
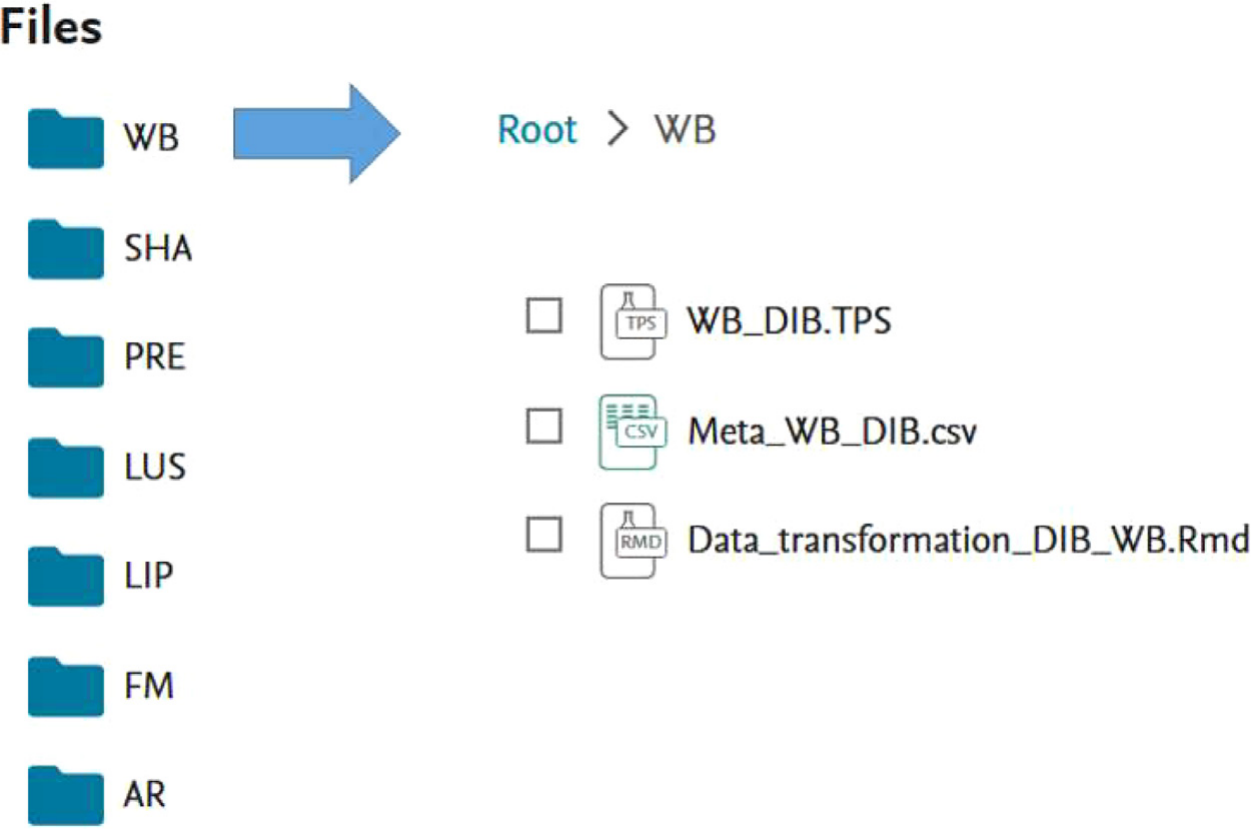
visual outline of the data repository.

On the repository, there is a folder for each breed containing the raw tps file of all digitized horses (including triplicates), and a metafile stored as a csv file (Fig. 1). We also provide the R code for data extraction and joint angle calculations from raw tps files, including indications on how to read the raw files into the script.

**Fig. 2:**
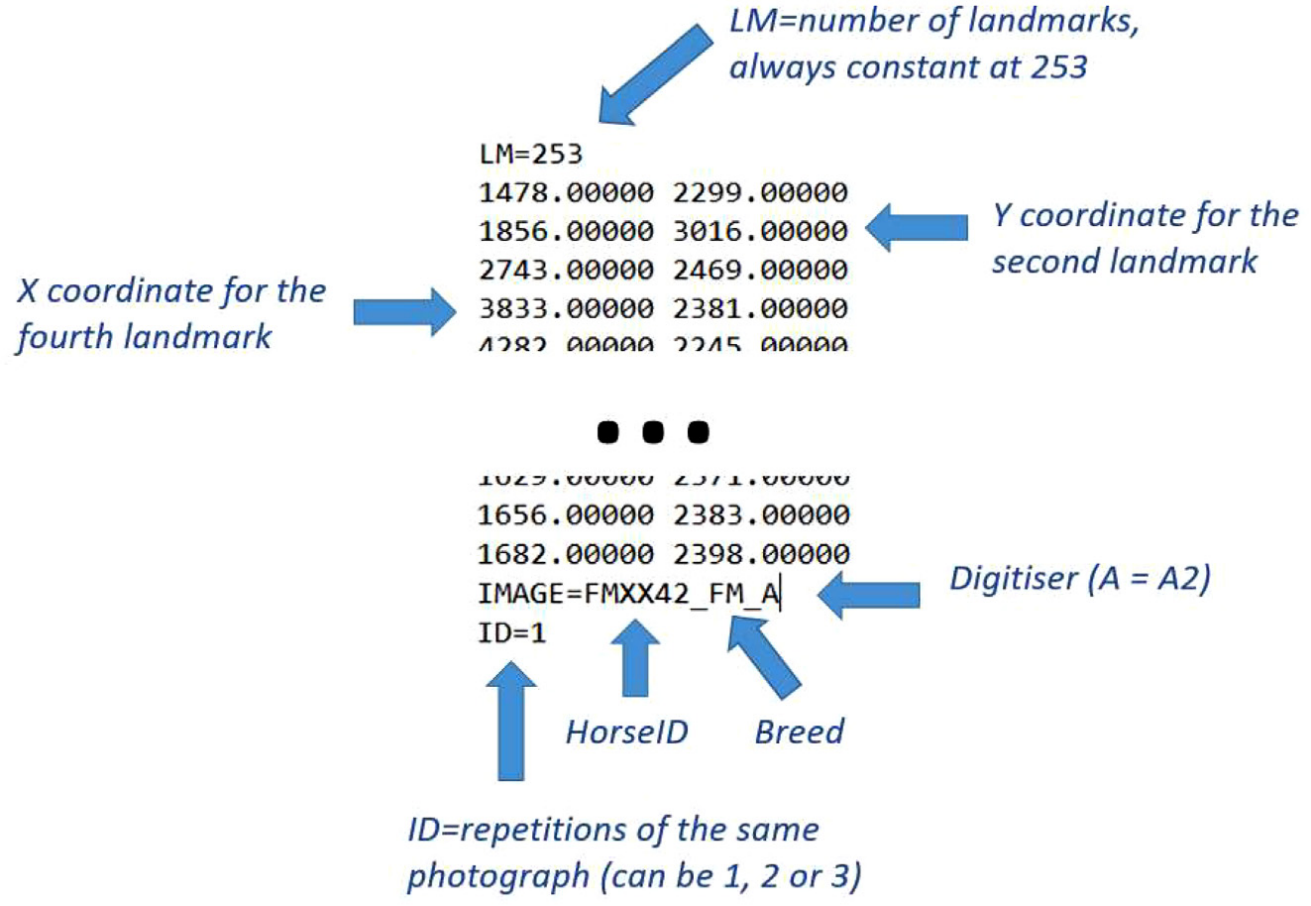
explanation on how to interpret the information in the tps files.

Each tps file starts with the number of lm present for each photograph, and ends with the information of whether it was the first, second or third digitization of the photograph (Fig. 2). Each row after “LM=253” is a lm coordinate (x on the right hand side, y on the left). The term “IMAGE=” represents the end of the coordinates and contains information on the individual (HorseID), the breed and the digitizer (A and A2 are equivalent). The term “HorseID” is the key linking the raw tps file to the metafile. Due to the HorseID separators being different for SHA and LIP (“-”) compared to the other breeds (“_”), we provide R markdown files for each breed separately.

The metafile starts with the individual horse identifier, followed by the sex, year of birth, year of the photograph to derive age on the photograph, wither's height in case someone wants to normalize the data to height, the country of origin of the photograph, and the scores for the six posture parameters previously described in Gmel et al. [4] (Table 1).

**Table 1:** list of variables included in the metafile. Posture variables were previously defined in Gmel et al. [4] and can be found in detail here: https://doi.org/10.1371/journal.pone.0202931.s005.

Variable	Definition	Abbreviation
HorseID	Individual horse identifier, key between raw data and metafile	–
Sex	Whether the horse is a stallion (male), gelding (castrated male) or a mare (female)	stallion = s gelding = g mare = m
Biosex	Whether the horse is biologically male or female	male = 1 female = 0
YOB	Year of birth of the horse	–
PhotoYear	Year the photograph was taken	–
Age	Age = YOB – PhotoYear	–
WH	Wither's height in cm	–
Country	Country where the photograph was taken	–
Head_height	How high the horse holds its head	1 = high to 3 = low
Head_camera	Whether the horse turns its head towards or away from the camera	from 1 = towards to 5 = away from the camera
Front_limb	Whether the front cannon bone is perpendicular to the ground, or the front limb is in front or behind the vertical axis	from 2 = behind the vertical axis to 5 = in front of the vertical axis
Hind_limb	Whether the hind cannon bone is perpendicular to the ground, or the front limb is in front or behind the vertical axis	from 5 = in front of the vertical axis to 1 = behind the vertical axis
Body	Whether there is one body part closer to the photographer in the photograph	from 1 = hind quarters closer to 5 = forequarters closer
Tail	Whether the tail was raised in excitement or not	1= relaxed tail 2= fully raised tail

In total, 1241 individual animals were included in the dataset, and 2489 photographs were digitized (Table 2). The photographs contained horses from seven breeds: Franches-Montagnes (FM), European Warmblood (WB), Lipizzaner (LIP), Shagya Arabians (SHA), Lusitanos (LUS), Purebred Arabian (AR) and Pura Raza Español (PRE). For WB and FM horses, many photographs were extracted from the archives of the Swiss National Stud Farm of Agroscope, with photographs dating back to the 1940ʼs. For the other breeds, we have obtained more contemporaneous photographs (between 2020 and 2023).

**Table 2:** Summary of available data. Number of animals (n), sex (stallions, geldings and mares), median year of birth (YOB), and mean age ± standard deviation (with missing data in parentheses).

Breed	n	Sex			YOB	Age
		s	g	m		
FM	742	526	102	114	2010	3.78 ± 2.40 (134)
WB	128	24	26	78	2013	7.78 ± 6.07
LIP	228	123	3	102	2005	10.42 ± 5.57
SHA	32	5	0	27	2010	5.67 ± 3.15
LUS	56	48	0	8	2014	9.82 ± 4.92
AR	34	10	1	23	2018	5.82 ± 5.46
PRE	21	19	1	1	2015	7.10 ± 2.99 (1)

Using the R codes provided in the repository, we also extracted the repeatability of the joint angles using intraclass correlation coefficients (ICC) and their 95 % confidence interval (Table 3).

**Table 3:** Intra-digitiser repeatability of the joint angles extracted from the raw landmark data according to Gmel et al. 2022 for each breed using intra-class correlation coefficients.

Joint angle	Abbreviation in code file	FM (485)	WB (21)	LIP (20)	SHA (32)	LUS (20)	AR (27)	PRE (19)
Poll	Poll	0.98 [0.98;0.99]	0.99 [0.98;0.99]	0.98 [0.96;0.99]	0.96 [0.93;0.98]	0.95 [0.92;0.98]	0.98 [0.97;0.99]	0.98 [0.97;0.99]
Neck-Shoulderblade	Neck	0.94 [0.94;0.95]	0.91 [0.83;0.96]	0.85 [0.73;0.93]	0.94 [0.90;0.97]	0.94 [0.87;0.97]	0.96 [0.93;0.98]	0.91 [0.83;0.96]
Shoulder joint	Shoulder	0.81 [0.78;0.83]	0.78 [0.62;0.90]	0.76 [0.57;0.89]	0.83 [0.72;0.90]	0.82 [0.67;0.92]	0.71 [0.53;0.84]	0.59 [0.34;0.80]
Elbow joint	Elbow	0.86 [0.84;0.88]	0.84 [0.70;0.92]	0.78 [0.60;0.90]	0.91 [0.85;0.95]	0.78 [0.61;0.90]	0.83 [0.71;0.91]	0.81 [0.65;0.92]
Elbow joint (in)	ElbowIn	0.86 [0.83;0.87]	0.84 [0.70;0.92]	0.76 [0.57;0.89]	0.90 [0.84;0.95]	0.81 [0.64;0.91]	0.81 [0.68;0.90]	0.82 [0.66;0.92]
Carpal joint	Carpus	0.43 [0.37;0.48]	0.64 [0.42;0.82]	0.58 [0.33;0.79]	0.45 [0.23;0.65]	0.68 [0.46;0.84]	0.34 [0.11;0.59]	0.39 [0.11;0.67]
Carpal joint (in)	CarpusIn	0.47 [0.42;0.52]	0.73 [0.53;0.87]	0.53 [0.27;0.76]	0.45 [0.24;0.65]	0.80 [0.64;0.91]	0.59 [0.38;0.77]	0.47 [0.19;0.72]
Fetlock joint front	FetlockF	0.66 [0.62;0.70]	0.84 [0.70;0.92]	0.62 [0.38;0.81]	0.81 [0.69;0.90]	0.66 [0.43;0.83]	0.86 [0.75;0.93]	0.80 [0.64;0.91]
Fetlock joint front (in)	FetlockFIn	0.74 [0.71;0.77]	0.80 [0.64;0.90]	0.75 [0.56;0.88]	0.91 [0.84;0.95]	0.90 [0.80;0.95]	0.90 [0.82;0.95]	0.85 [0.71;0.93]
Hip joint	Hip	0.90 [0.88;0.91]	0.92 [0.85;0.96]	0.85 [0.73;0.93]	0.82 [0.71;0.90]	0.92 [0.84;0.96]	0.90 [0.82;0.94]	0.93 [0.87;0.97]
Hip joint (in)	HipIn	0.92 [0.91;0.93]	0.93 [0.86;0.97]	0.90 [0.81;0.96]	0.90 [0.83;0.95]	0.89 [0.79;0.95]	0.94 [0.90;0.97]	0.85 [0.71;0.93]
Stifle joint	Stifle	0.88 [0.87;0.90]	0.87 [0.75;0.94]	0.91 [0.81;0.96]	0.79 [0.66;0.88]	0.87 [0.74;0.94]	0.86 [0.76;0.93]	0.89 [0.79;0.95]
Stifle joint (in)	StifleIn	0.89 [0.87;0.90]	0.77 [0.59;0.89]	0.90 [0.80;0.96]	0.89 [0.81;0.94]	0.88 [0.77;0.95]	0.91 [0.83;0.95]	0.85 [0.71;0.93]
Hock joint	Hock	0.82 [0.80;0.85]	0.84 [0.70;0.92]	0.94 [0.88;0.97]	0.86 [0.77;0.93]	0.87 [0.76;0.94]	0.91 [0.84;0.95]	0.75 [0.55;0.88]
Hock joint (in)	HockIn	0.78 [0.75;0.81]	0.73 [0.54;0.87]	0.92 [0.85;0.97]	0.90 [0.83;0.94]	0.76 [0.58;0.89]	0.93 [0.87;0.96]	0.79 [0.61;0.90]
Fetlock joint hind	FetlockH	0.77 [0.74;0.80]	0.88 [0.77;0.94]	0.81 [0.66;0.91]	0.84 [0.74;0.91]	0.61 [0.37;0.80]	0.92 [0.85;0.96]	0.82 [0.66;0.92]
Fetlock joint hind (in)	FetlockHIn	0.82 [0.79;0.84]	0.88 [0.78;0.95]	0.86 [0.74;0.94]	0.85 [0.75;0.92]	0.78 [0.61;0.90]	0.91 [0.83;0.95]	0.88 [0.76;0.95]

## 4 Experimental Design, Materials and Methods

**Fig. 3:**
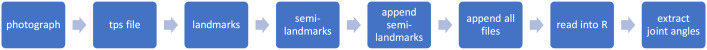
Summary of data processing procedures.

For each individual horse, we selected the photograph in which the horse was closest to the ideal posture proposed by Druml et al. [5]: “[the horses] were […] captured in the so-called ‘open posture’, where the left foreleg stands vertical, the hoof of the right foreleg is located one to two hoof lengths behind the left foreleg, the cannon bone of the right hind leg is near the vertical and the hoof of the right hind leg is located two to three hoof lengths before the left hind leg. Neck and head should be presented in a natural way”. Once the photograph was selected, we created a tps file linked to the photograph using the program tpsUtil v1.78. We then proceeded to the digitization using tpsDig v2.16 (Fig. 3). The exact lm placement was described in Druml et al. [5], Gmel et al. [4] and Gmel et al. [1].

**Fig. 4:**
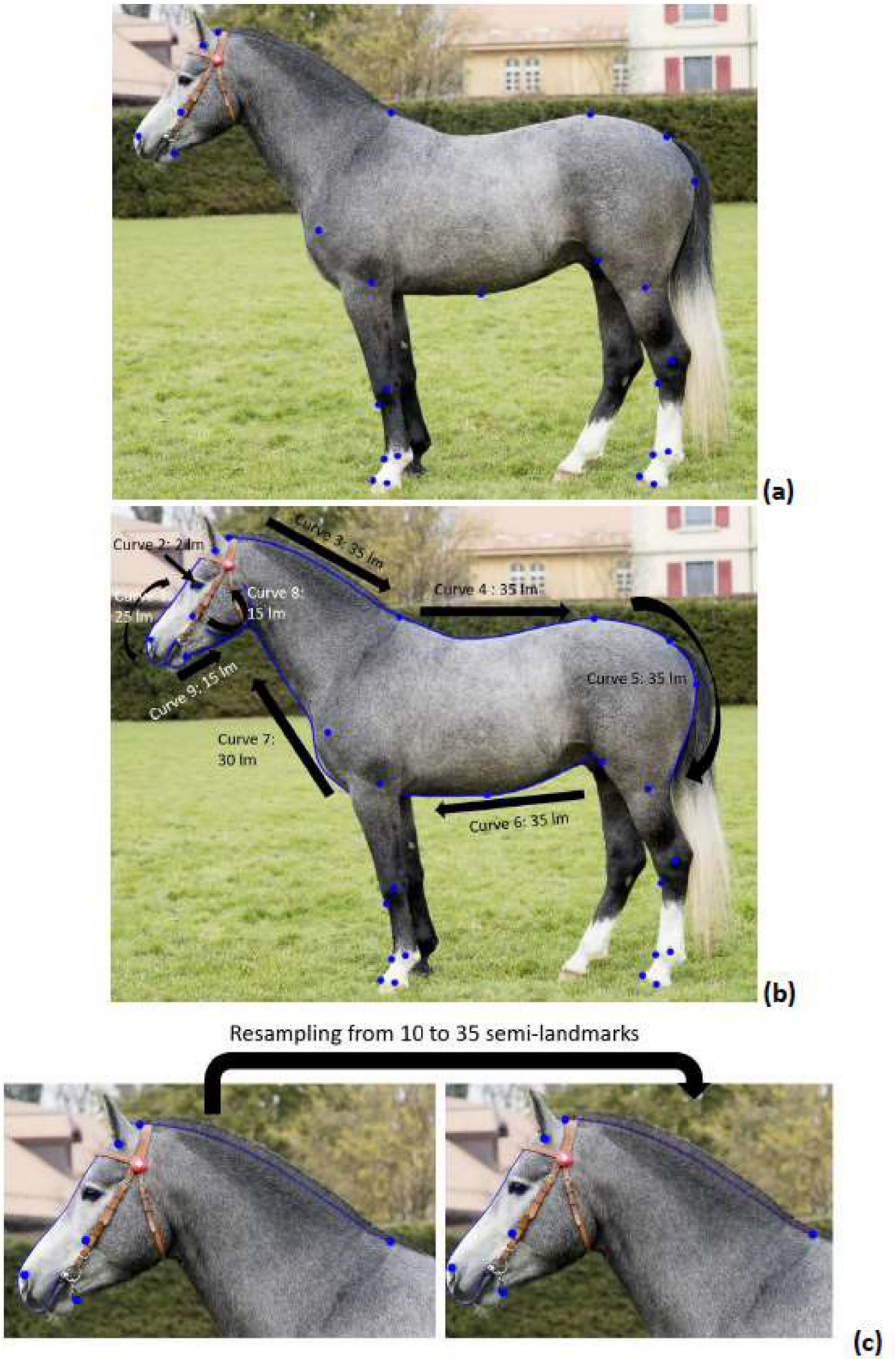
methodology for the landmark placement. In the first step (a), only the landmarks are placed. In the second step, the curves are traced (b). Each curve is resampled for the right number of landmarks that are placed equidistantly (c).

**Fig. 5:**
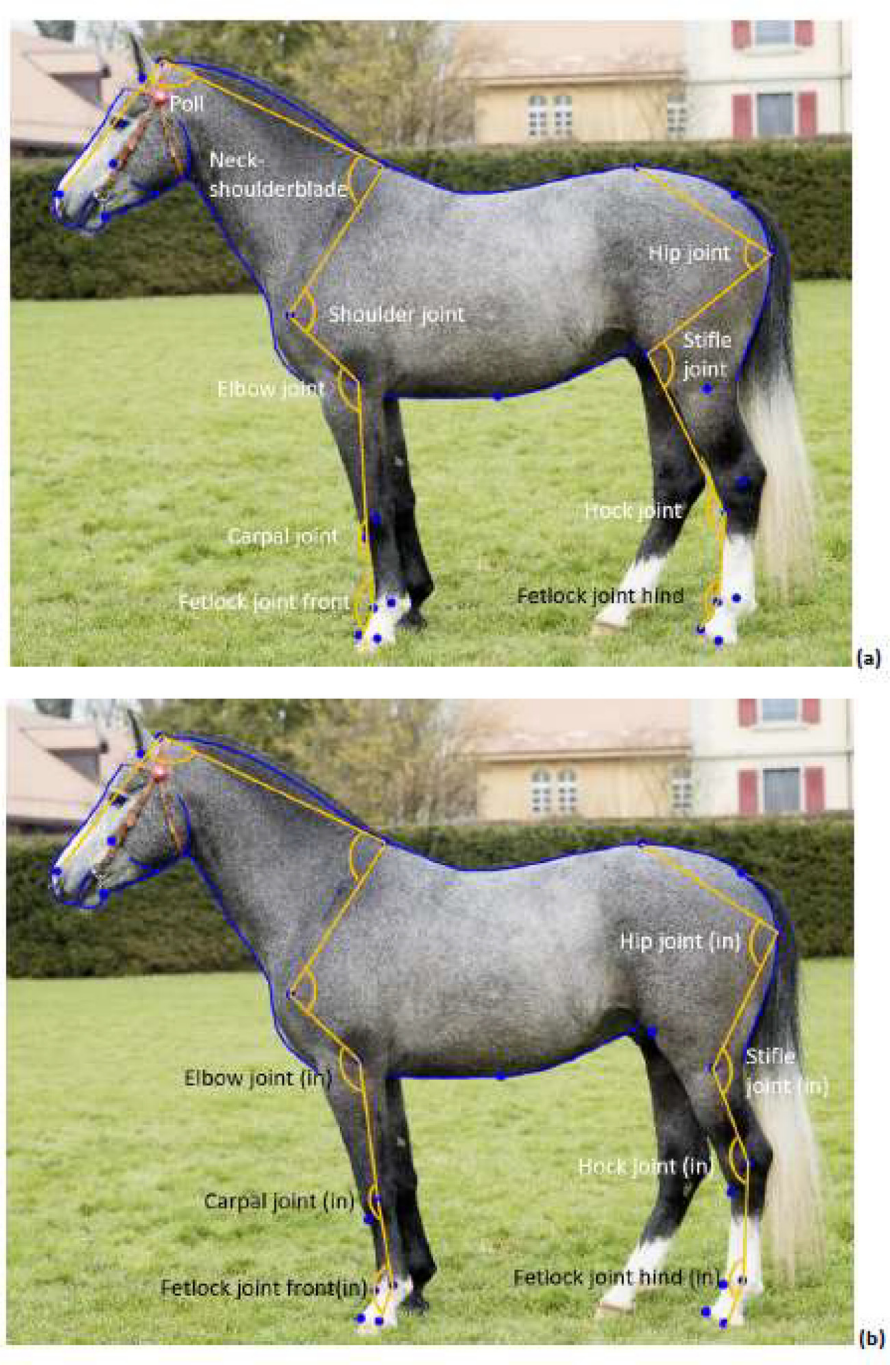
location of the joint angles, placed outside (a) or inside the joint (b).

In a first step, we placed the full 26 lm that are also the basis for the joint angle measurements (Fig. 4A). We then traced the curves between the lm (Fig. 4B). Curve 1 traces the outline of the nose and forehead with 25 slm. Curve 2 consists of only two slm in the corners of the eye. Curve 3 traces the shape of the neck (35 slm), curve 4 the shape of the back up to the highest point of the croup (35 slm), and curve 5 the shape of the croup (35 slm). Curve 6 traces the underside of the abdomen (35 slm), curve 7 the underside of the neck (30 slm), curve 8 the cheek (15 slm) and curve 9 the underside of the jaw (15 slm). The number of slm was determined by Druml et al. [5] to best cover the outline of each curve while having enough space to place all the slm. Therefore, the smaller curves (curve 8 and curve 9) have fewer slm than the longer and more bent curves of the neck and back (e.g. curve 3 and 4). After an initial fit, the semi-landmarks were resampled by a constant distance along the curve (Fig. 4C). The semi-landmarks were appended to the rest of the landmarks in a new tps file. The new tps files were merged into the final file provided on the server. The final file was read into R using the code provided in the .rmd file to extract joint angles (Fig. 5).

## Limitations

The major issue during data collection was the posture of the horse, often associated with the excited status of the horse. This has partially been addressed by classifying the posture of the horse on the photograph, with scores available in the metadata. The other issue is the unequal sample size, due mainly to limited access to some breeds compared to others.

## Ethics Statement

Many of the photographs originated from archives or were provided by owners. Some photographs were taken during experiments under animal permit numbers VD3096 or VD3527b. No animal was harmed or unduly solicited over their coping capacity during the experiment. The original photographs are not provided to ensure anonymity of the horses, handlers and owners. The experiments complied with ARRIVE guidelines.

## CRediT Author Statement

**Annik Imogen Gmel**: Conceptualisation, Methodology, Formal analysis, Investigation, Data curation, Writing –original draft, Visualisation, Funding acquisition; **Monika Stefaniuk-Szmukier**: Investigation; **Weronika Klecel**: Investigation; **Tania Martin-Gimenez**: Investigation; **Antonio Cruz**: Investigation; Luis P Lamas: Investigation; **Teresa V Rosa**: Investigation; **Michael Andreas Weishaupt**: Conceptualisation, Methodology, Investigation, Data curation, Writing –review & editing, Resources, Supervision; **Markus Neuditschko**: Conceptualisation, Methodology, Writing –review & editing, Funding acquisition, Resources, Supervision, Project administration.

## Data Availability

TPS coordinate files of horse shape space data (Original data) (Mendeley Data).
